# Oral microbial interactions from an ecological perspective: a narrative review

**DOI:** 10.3389/froh.2023.1229118

**Published:** 2023-09-13

**Authors:** Daniel H. Fine, Helen Schreiner

**Affiliations:** Department of Oral Biology, Rutgers School of Dental Medicine, Newark, NJ, United States

**Keywords:** ecology, caries, periodontitis, biofilms, microbiome, fitness

## Abstract

Landscape ecology is a relatively new field of study within the sub-specialty of ecology that considers time and space in addition to structure and function. Landscape ecology contends that both the configuration (spatial pattern) and the composition (organisms both at the macro and or micro level) of an ecology can change over time. The oral cavity is an ideal place to study landscape ecology because of the variety of landscapes, the dynamic nature of plaque biofilm development, and the easy access to biofilm material. This review is intended to provide some specific clinical examples of how landscape ecology can influence the understanding of oral diseases and act as a supplement to diagnosis and treatment. The purpose of this review is two-fold; (1) to illustrate how landscape ecology can be used to clarify the two most prominent microbiologically induced infections in the oral cavity, and (2) how studies of oral microbiology can be used to enhance the understanding of landscape ecology. The review will distinguish between “habitat” and “niche” in a landscape and extend the concept that a “patch”, is the demarcating unit of a habitat within a landscape. The review will describe how; (1) an individual patch, defined by its shape, edges and internal components can have an influence on species within the patch, (2) spatial dynamics over time within a patch can lead to variations or diversities of species within that patch space, and (3) an unwelcoming environment can promote species extinction or departure/dispersion into a more favorable habitat. Understanding this dynamic in relationship to caries and periodontal disease is the focus of this review.

## 1. Introduction

This overview is written with two audiences in mind; oral health professionals, and landscape ecologists who focus on microbiology. For oral health professionals this review is designed to act as an introduction to landscape ecology and how it can relate to the diagnosis and treatment of dental diseases. For landscape ecologists, who have a limited background in oral health, this overview will describe the unique features of oral microbial patterns as they relate to the two most prevalent dental diseases, caries and periodontitis (1, 2). For purposes of clarity the portion of the review that focuses on landscape ecology will be presented in three boxes, while the portion devoted to health professionals will be presented in the text. Landscape ecology will be organized into; (1) definitions and theories (Box 1), (2) historical aspects of ecology (Box 2) and community ecology (Box 3). The oral health professional portion will be divided into 4 sections related to caries and periodontal disease. The goal of this review is four-fold, (1) to illustrate how ecological principals can enhance our understanding of oral microbiology, (2) to describe how ecological principals have been used in dentistry in the past, (3) to show how commensals in the oral environment can disperse to distant organs and exacerbate disease at these distant sites such as the heart, kidney and colon, and (4) to describe how studies of ecological principals of the oral microbiome can lead to a better understanding of landscape ecology that can potentially evolve into new diagnostic and therapeutic strategies for oral and systemic health.

BOX 1 **Definitions and Theories of Ecology.**

Our initial effort to clarify ecological terminology will be to distinguish between a “habitat” and a “niche” as they relate to caries and periodontal disease, using microbiological examples to illustrate how these diseases can be influenced by the landscape they live in (17). The habitat can be thought of as the ‘Home”, or the address of the microbes. The work done in the habitat, or the role the microbes play in that habitat can be described as its niche (2, 18). A home or habitat can house several niche activities. A variety of physical (abiotic) and biological (biotic) conditions can occur within the habitat that support growth and survival of microbes within that habitat (19, 20). A niche can be described as how a microbes activity fits into its home. A basic sub-unit of a landscape is referred to as a “patch”. A “patch” is typically depicted as a significant part of a habitat. A “patch” can demonstrate unique features that differentiate one area from another within the overall landscape. To better define the dynamics within a patch or between “patches” it is beneficial to describe a patch in terms of space, time, structure, and function (18, 19).

To illustrate how these terms relate to biology, an aquarium can serve as an example of a home to many species related activities depending on the pH, temperature, oxygen levels, minerals etc. and the biological and abiotic materials within that aquatic habitat (21). The habitat, in this example an aquarium, can support the growth of plants, fish, or combinations of these (22). The size of the of the water body among many other characteristics such as food availability and type as well as competition among inhabitants can determine the residents that live in the aquarium (8, 23, 24). To illustrate this point, a small common goldfish (*Carassius auratis*) in an enclosed aquarium could be much larger in a lake. It has been shown that when the goldfish is grown in a small tank γ-aminobutyric acid (GABA) is produced by the resident goldfish and the GABA inhibits the secretion of the growth hormone “somatostatin” that suppresses the growth of goldfish resulting in a “small fish in a small pond” as opposed to a “big fish in a small pond” (25). This signaling resembles quorum sensing between bacteria where Gram+ bacteria use acylated-homoserine-lactones, and Gram− bacteria use processed oligopeptides, to communicate (26). Obviously, food availability and competitors, both quantity and quality, will support niche activities and growth and survival in either environment but this intercellular communication has been thought to have been the evolutionary spark that initiated multicellular development (27). Thus, the habitat, its shape and content, are important determinants that can have a direct influence on how species activity fits into the environment, i. e., the flow of energy or metabolites from one microbe to another and/or the signal sent from one microbe to another (17, 24). One specific example features the LuxS/AI-2 system (28). Overall community activities in many ways are defined by the habitat, its boundaries, size (small or large) and contents (food or metabolic elements provided) (21, 29). Plainly put, a research laboratory (habitat) could contain; an electron microscope (for cell biology), a HPLC instrument (for biochemistry), and an anaerobic chamber (for microbiology) and can be thought of as a multidimensional/multifaceted environment with the same “home”, but several niches with different activities. Coordination of these activities can be accomplished by communication (i.e., the LuxS/AI-2 system) in this case by the laboratory director to his or her associates (29).

BOX 2 **Ecology: Historical Beginnings.**

In the earliest days of ecology, the principal determinant of mammalian, avian and fish species was initially thought to be due to place (i. e., geography), although reference to function within place was discussed (36). Biogeography was considered to be the central feature of the evolution of species, and it was proposed that environments that were similar but were geographically separated showed differing species diversities (37). This theory was championed by Linnaeus in the 1700's favoring environmental determinism (36). Darwin (38) and AR Wallace (39), believed in a stable biogeography but that dispersal from one place to another was possible (38, 39). However, mammalian species were thought to be found because of unique geographies that were determined when tectonic plates either diverged, converged, or moved laterally and Pangaea or Gondwanaland was divided into different continents (40). Thus, kangaroos were found in Australia and landmass separations were the ultimate determinant of where species were discovered. It was proposed that physical boundaries created by landmasses separated by water could only be overcome by island hopping, and this species re-location was only possible when the island separation was narrow and shallow making island hopping easier. The study of microbiology was at that time in its infancy (41).

The understanding of ecology has become significantly more multifaceted, and factors such as competition, climate, food, etc., add to this complexity (42). This initial biogeographical theory has been challenged to the greatest extent by the theory that “everything is everywhere but the environment selects” (43). This “environmentally based” theory is of paramount importance to infectious disease specialists and a current example of this environmental selectivity is seen in SARS CoV 2 which can be transmitted worldwide but requires a specific receptor/adhesin interaction for disease to occur (27, 44).

BOX 3 **Community ecology and futuristic visions.**

Community ecology can change due to deterministic influences. Selection within a niche initially due to attachment properties can cause a significant change in the local community composition (120). A low pH or elevated oxygen content or metabolites can force microbes that cannot survive to flee (121). These deterministic influences can force dispersal and movement away from challenges they face in one patch and force them to move to a more accommodating patch (121). These changes need to be studied in an environment that shows *in vivo* relationships that relate to metabolomics and proteomics seen in the oral cavity. Changes can also occur due to evolution (speciation) making the new species more fit to occupy the vacant habitat space (122). The Environmental Plague Hypothesis was prescient in this regard (122). Once again tissue conditions are most likely related to metabolites available for growth and survival. Speciation can result in acquisition of new genes to accommodate for; (1) metabolite availability, or (2) adhesin/receptor specificity), or (3) stochastic or random influences (such as extinction of a species forming a void permitting overgrowth of another opportunistic species to fill that void) (123, 124).

Many niche-based theories imply that selection is critical in the early stages of community ecology while dispersal is more deterministic in the later stages of community development. This is not universally accepted since both drift and dispersal can occur in open habitats where community extinction provides for an opportunity for new species to either disperse or drift into niches that fit their needs (125). Dispersal typically is due to deterministic movement, such as local H_2_O_2_ hazards whereas drift appears to occur in a random manner into communities where competitiveness is minimal, and edges and barriers are weak or absent (126, 127). While these complex determinants are ever changing, we can be sure of the fact that bacteria due their adaptability and short generation time have the best chance of surviving catastrophic events. In Francis Crick's book “Life Itself” he projected the possibility of earth as a failing planet due to insufficient energy from the sun resulting from a climate crisis (128). Musing on how scientists could maintain a sustained life form on a new planet in a new universe, he proposed that we could use rocketry to deliver capsules filled with diverse species of bacteria to “everywhere”. This theory of “directed panspermia”, was his hope of creating a “new beginning” by having rockets land in an atmosphere that would provide the appropriate environmental conditions supporting growth of one microbial species that would allow life to begin again. On a somewhat less “other worldly” approach, Martin Blaser in “Missing Microbes” has proposed that one goal would be to collect and then “bank” microbes obtained from indigenous populations who have not been exposed to antibiotics (129). This “bank” could be used to restore missing microbes and thus reconstitute our ‘natural microbiome” required to protect humanity from uncontrolled/uncontested infectious overgrowth. While Crick's vision is fully acknowledged as science fiction, the Blaser vision is more immediate.

The principal challenge and the focus of this overview is how the concept “everything is everywhere but the environment selects” could be valid in the oral cavity (3, 4). This is particularly testable for microbes that find their way to the oral cavity where the mouth is the entryway to a multitude of agents but ultimately embraces a select group of specific taxa, while most of the elements that enter the oral cavity are transient (1, 5, 6). This review will center on key oral diseases caries and periodontal disease. Since our focus is on oral microbiology, the main contention regarding oral microbiology is that one oral habitat can house a niche community with a unique set of microbial species that will respond to available resources within that habitat differently than another set of microbial species (7). Communication between species is another critical and complex overall niche activity determinant (8). LuxS is a well-studied signal protein precursor that produces an autoinducer-2 (AI-2) molecule utilized for communication by many biofilm formers that span both Gram+ and Gram− phenotypes as well as fungi (9). The LuxS/AI-2 system is purported to regulate biofilm community behavior especially cell density (10). To reinforce the notion that communication is important but complex, *in vitro* monoculture experiments show that a *luxS* gene deletion results in loss of the AI-2 signal but biofilm density in that monoculture can still occur, albeit at lower density due to upregulation of compensatory adherence and extracellular polymer genes in the LuxS deleted species (11, 12). However, *in vivo* multicellular biofilm experiments indicate that LuxS absence in one biofilm member is compensated for by overexpression of other AI-2 signaling molecules produced by other community members (12). Therefore, since the QseB, LsrB, and/or the RbsB receptors are still intact, the AI-2 signal can come from other community members and replace a luxS deletion in the mtutated strain (12). These results point to the importance and complexity of the cell to cell signaling relative to the modulation of cell density and niche activities.

In spite of these compexities, these signaling and/or community niche activities, if understood, could be a significant determinant for either a benefit or detriment to the host and could alter the shape/structure or signaling within a particular habitat (13). Novel modulation of cell to cell signaling could provide a future method of attempting to alter biofilm habitat composition. Although habitat alteration has not been stated as an overriding scientific principle of dental treatment, many treatments dating back to G.V. Black's “extension for prevention” were designed to alter the local/dental environment such that dental restorations could be extended to self-cleansing areas (14). This strategy resulted in retarding re-emergence of bacteria responsible for decay (14, 15). Undoubtedly, microbial community adaptability in a specific oral habitat whether related to disease or health at the local oral level will dictate the prevailing balance between a “damage” and/or a “health response” (16). This review will illustrate how habitat selectivity can alter health or damage related to oral bacteria.

## 2. The oral cavity an area to utilize for studies of landscape ecology

The contention of this overview is that the oral cavity, with all its discreet variable regions, can serve as an ideal research environment for the study of landscape ecology (2, 19). Within the oral cavity, regions that provide a rich and unique habitat available for study include, but are not limited to, areas such as the dorsal and lingual surfaces of the tongue, the buccal soft tissue, the palatal tissue, the various tooth surfaces (occlusal, proximal, root surface, buccal and lingual) as well as areas above and below the gum-line (2, 19). Each of these regions, from the point of view of microbial habitats, has its own spatial patterns (i.e., shape, boundary, and edge), its own activity that occurs within (that space), and its own potential for a shifting microbial population that can effectively change biogeographical outcomes (1, 30). From a microbial fitness point of view, the microbe can be a specialist or a generalist (31). A specialist requires a specific set of factors to support its fitness (pH, aerobiosis, temperature), while a generalist shows a much greater adaptability and can adjust to a variety of conditions. Overall, using microbes to study landscape ecology has several advantages as follows; (1) microbes have a rapid generation time, (20 min to several hours to days), (2) microbes are easily accessible, (this is especially true of oral microbes which can be collected without any invasive procedures), and (3) microbes in the oral cavity can be studied overtime without causing any major irreversible health implications (32). Another benefit of use of the oral cavity as a hallmark of ecological studies is that new methodologies can allow for a full visualization of biogeographic relationships that can affect a specific habitat or region (tongue, cheek, tooth surface) (33–35).

The enamel and periodontal tissue habitats will serve as examples of the four principals of community ecology, (a) microbial selection, (b) speciation, (c) dispersal, and (d) drift. From a landscape ecology point of view, it will be instructive to define enamel caries according to the habitat and niche of the infectious assault (2, 5) and as such caries will be separated into occlusal and smooth surface caries. Dental Caries will be used as abroad term employed to identify an enamel lesion that results from oral biofilms that accumulate adjacent to the tooth surface and that cause demineralization of the enamel surface. Under the appropriate conditions of frequent ingestion of carbohydrates, the tooth-related biofilm can produce a pH that drops below 5.5 and overtime (45), after repeated exposure to these carbohydrates' acid removal of calcium mineral from the adjacent hydroxyapatite crystal structure of the enamel occurs (45, 46). This oversimplified definition of caries fails to differentiate between occlusal and smooth surface caries but also notably ignores root caries that results from demineralization as well as protein degradation of the root surface composed of a calcium infused collagen matrix (47). This often-used definition of caries fails to identify how the habitats and niches differ in these aforementioned three caries disease entities (occlusal, smooth surface and root caries) and importantly misses ways in which defining the habitat and niche can likely lead to new strategies in treatment and prevention of disease in these areas. While altering the habitat is not a new approach to dental treatment (48, 49), the concept has not been re-enforced as a theory for diagnosis and treatment, and if used in this manner, oral health professionals can alter the niche activity and can cause a shift away from damage by pathobionts in favor of response and repair by growth of commensals (50).

For purposes of illustration occlusal and smooth surface proximal decay will be described below.

## 3. How landscape ecology can provide a better vision of dental caries

**Figure 1:**
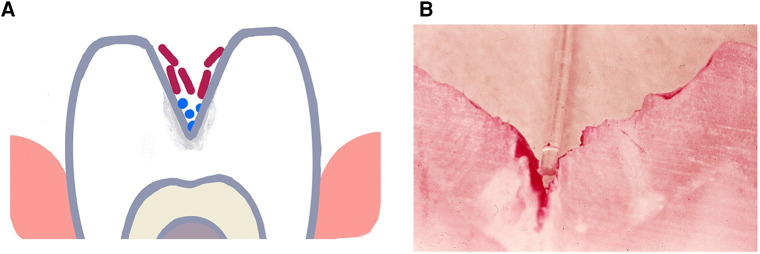
Illustration of a deep torturous fissure that occurs on molar surfaces and allows for accumulation of non-adherent bacteria such as lactobacilli spp. in the fissure (**A**). Here the microbe can produce acid, cause demineralization of the enamel and create an inaccessible carious lesions (**B**). Cross-section of a molar with a deep fissure showing a tooth-brush bristle placed in the fissure above dental plaque stained red in the depth of the fissure with demineralized enamel below the plaque.

In spite of the fact that, the end result of either form of enamel caries is demineralization, separating occlusal and smooth surface caries provide an excellent example of how a habitat and the contents of that habitat create a niche that can select for specific microbial types that can influence diagnosis, treatment and prevention of disease. Occlusal caries occurs in the pits and fissures of the occlusal surface of teeth. As seen in Figure 1 organisms get physically impacted into pits and fissures that occur on occlusal surfaces of molars and premolars and are found in tortuous sites that are somewhat shielded from mechanical and perhaps even chemical intervention (46, 51). These protected sites do not require microbial colonizers to possess any adhesin/receptor binding specificity for retention in these labyrinthian regions (52, 53).

Enamel caries occurs because of the ability of a microorganism or a consortium of microorganisms to produce acid (acidogenic) over a specified spatial location, over a repeated time, and tolerate living in an acid environment (aciduric) (54, 55). These qualities are mandatory, and typically these microbes are embedded in an extracellular polysaccharide matrix that forms a gelatinous biofilm adjacent to the enamel that was initially called dental plaque, now referred to as a dental biofilm (22, 45). The matrix can prevent the bacteria from being washed away by saliva and can also act as a passageway for salivary modification of the biofilm by buffering or antimicrobial activity (56, 57). To produce enamel demineralization, however, these bacteria require the capability of producing acid, in many cases lactic acid (58). Whereas, early culture-based studies of caries sited, Lactobacillus spp., and *S. mutans* as the primary causative agents, newer molecular methods have expanded caries associated bacteria to include; Veillonella spp., Leptotrichia spp., Actinomyces spp., Bifidobacterium spp., Propionibacterium spp., *Scardovia wiggsiae*, Atopobium spp., and Candida spp. (22, 59, 60).

### 3.1. Influence of host microenvironment on spatio-temporal modulation of community dynamics in caries

Clearly salivary flow and content as well as mucin composition can modulate microbial community dynamics (61). Both immune associated and immune-independent salivary factors are involved (61). Microbiome regulatory immune related salivary components such as; lactoferrin, lysozyme, lactoperoxidase, and IgA modulate community life (57). Further, mucins derived from non-immune related cells can also play a distinctive role in adherence/clearance ratio of supragingival bacterial communities (62). Moreover, as will be described below, both host and microbial factors can have a major impact on another paramount dental disease, periodontitis (63).

### 3.2. Influence of the habitat and niche on diagnosis and treatment; occlusal caries

Of particular relevance to this review are studies by Dige et al., 2020 using Fluorescent *In Situ* Hybridization (FISH) showing Streptococcus spp., Actinomyces spp., Lactobacillus spp., and Bifidobacterium spp., in pits and fissures of demineralized occlusal carious lesions (44, 64). In this case the anatomy of the pit or fissure provides a home for bacteria that can actively survive in a protected site without having to possess adhesive factors. Therefore, this torturous anatomy obviates the need for the acid producing bacteria to supply its own adhesins (51, 52). Strategies to modify these torturous landscapes over the years have included; (1) application of silver nitrate to infiltrate the deep fissures to kill the microbes and prevent caries (48) including site directed application to deep pits and fissures for annihilation of these microbes (65), (2) prophylactic odontotomy to modify the deep fissures into smooth accessible, cleansable areas (66), (3) use of amalgam to fill or eliminate the pits and fissures after surgical enlargement and (67) and most recently (4) use of plastic sealants to fill the fissure and smooth them out (Table 1) (51). These measures have one thing in common they make-an-effort to change the pit and fissure anatomy to make it less torturous (68), less fissure-like and less likely that microbes can use the original habitat to find fitness in a niche that permit the microbes to multiply, produce acid and cause decay (50).

**Table 1:** Ecological determinants related to dental diseases.

Ecological terminology	Defined area	External influences	Evolving concepts	References
Habitat	Home	pH, temp, oxygen, nutrients	Geographic boundaries	(23, 45, 54, 121)
Patch	Portion of the home	Same as above	Environ selects	(3, 17–19)
Niche	Activity that occurs in any portion of the home	Signals, quorum sensing, etc.	Everything is everywhere but the environment selects	(8–10, 21–26)
Microbial characteristics	Flexible/rigid	Nutrition and environment	Stage of disease	
Specialist	Strict requirements for survival	Restrictive temp., oxy, nutrition	Later stages of damage/response	(1, 2, 16, 18, 27)
Generalist	Highly adaptable	Pioneers; flexible regarding above	Early stages of damage/response	(16, 40, 43, 45, 46)
Advantage of oral microbiome	Generation time	Accessibility	Collection invasiveness	
	Rapid generation time	Accessible for study	Collection with no health implications	(5–7, 24, 32, 52, 59)
Dental condition	Landscape	Initiating microbial factors	Treatment	
Caries: occlusal	Pits and fissures of occlusal surfaces	Acid production	Extension for prevent silver nitrate odontotomy Odont and amalgam sealants	(48, 49, 51, 53, 54)
Caries: proximal	Smooth surfaces of teeth	Acid production	Antimicrobial rinses physical plaque removal enzymes passive immunity	(52, 53, 70–78)
Periodontal microbiome:	Landscape	Initiating microbial factors	Treatment	
Supragingival	Saliva and pioneer colonizers	Carbohydrates	Brushing and antiseptics	(83–86, 88–91, 122)
Subgingival	Serum and migrating supragingival components	Proteins and small chain fatty acids	Neosalvarsan tetracycline and CHX vaccines	(83, 105–112)

### 3.3. Influence of the habitat and niche on diagnosis and treatment; smooth surface caries

**Figure 2:**
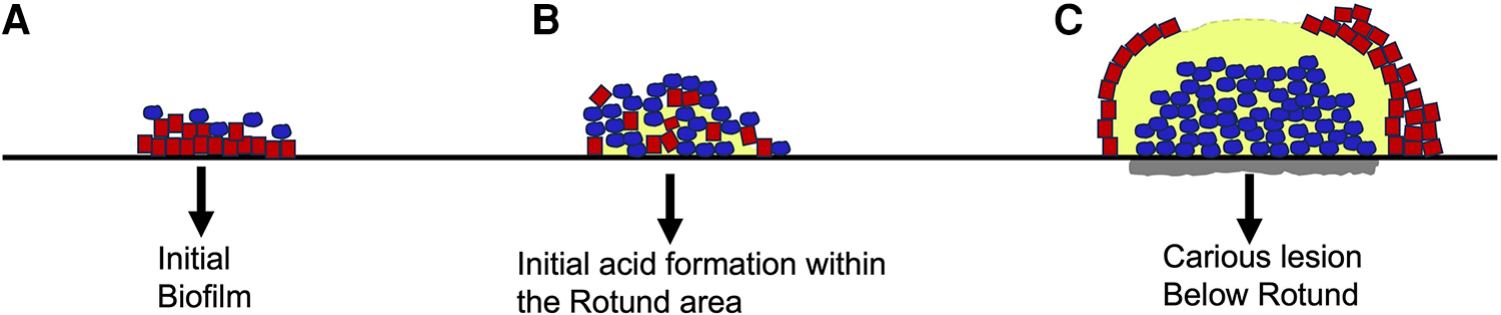
Architecture of S. mutans biofilm development overlying an enamel slab in vitro. Initial biofilm colonization showing S. mutans (in blue) on the outer surface of the biofilm. Line below red colonies is unaltered enamel slab (**A**). Intermediate biofilm showing S. mutans colonies expanding and moving closer to enamel slab. Acid release begins as “rotund” or patch begins to develop (**B**). Fully developed “patch” or rotund with a centered concentration of acid resulting in demineralization of slab due to overgrowth of S. mutans (grey area below blue colonies is S. mutans “rotund”) (**C**). Modified from reference (60).

Smooth surface caries occurs on the buccal, lingual, and proximal surfaces of the tooth. For these surfaces to be colonized microorganisms need to form a biological bond with material on the tooth surface (60). This boundary between the microbe and the salivary coated tooth surface occurs via electrostatic, physicochemical, and biological interactions typically requiring adhesin/receptor contact for prolonged associations (52, 57, 69). While lactobacilli are routinely found in occlusal pits and fissures, they lack surface adhesive properties, and require a retentive area (62). Lactobacilli spp. also thrive at a low pH and require access to carbohydrates (45, 52, 70). Lactobacilli are not found as pioneer colonizers and are considered by many as opportunistic microbes. In contrast, *S. mutans* has many attributes (adhesins, extra-cellular polysaccharides, glycosyl binding proteins) that enable it to adhere to smooth surfaces (71). Moreover, sucrose metabolism by *S. mutans* results in a thick sticky polymeric extracellular polysaccharide, glucan, that tends to bind the cells together to form a protective shield (72). *S. mutans* is not a pioneer colonizer of enamel but in the presence of sucrose with the production of a *S. mutans* derived gelatinous glucan coupled with fimbrial extensions, other adhesins such as Antigen I/II, Glucosyl transferase, the combination of these factors makes *S. mutans* a formidable early tooth colonizer (45). *S. mutans* is both acidogenic and aciduric and thus, once it has colonized, and the pH of the environment changes, *S. mutans* has an ecological advantage as compared to other acid vulnerable microorganisms. As mentioned, Streptococci spp. for the most part do not require retentive surfaces. In many ways, *S. mutans* is more adaptable than Lactobacilli spp. and thus can be considered more of a generalist. Damage to the smooth surface of enamel and dentin can change the local environment to one that is favorable to non-adherent bacteria and thus permit lactobacilli spp., a minimally adaptable specialist type microbe to gain a foot-hold in retentive spaces (52). Recent studies indicate that *S. mutans* produces an extracellular scaffold that forms a rotund structure with an inner *S. mutans* core. This core “patch” district is surrounded by non-mutans microbes and the patch can be located directly above a demineralized enamel region Figure 2 (60).

### 3.4. Influence of the habitat and niche on treatment modalities

While pit and fissure environments can be altered by sealants, smooth surfaces can also be altered. In this case, *S. mutans* can colonize in the interproximal area below the contact points of two adjacent tooth surfaces making it difficult to remove the biofilm by physical means. Flossing, a physical method of biofilm removal is difficult while antimicrobial rinsing has proven to be somewhat effective and trials have been conducted using chlorhexidine or other antimicrobial rinses and varnishes to interfere with microbial attachment capacity (72, 73). This method plus frequent professional cleanings have been used quite successfully in public health efforts in Sweden in the past (74, 75). Other smooth surface altering methods include, (1) enzymatic effects on salivary proteins (76), (2) antiseptic treatment of tooth surfaces to prevent or reduce attaching bacteria (74, 75), (3) passive application of antibodies to tooth surfaces to block adhesin receptor interactions (77), and (4) as mentioned physical removal of plaque by brushing and flossing. Local application of plant developed antibody to *S. mutans* Antigen I/II has been successful in showing a reduction in primate recolonization of *S. mutans* as a passive immunization strategy (78) (Table 1). These studies while showing modest success in changing the local environment have not been applied to practice more than likely because of cost-benefit ratios. Nevertheless, these studies provide additional evidence that alteration of the environment can alter disease. Unlike the carious process where there is a direct effect of biofilm acid production on tooth enamel resulting in demineralization, periodontal disease is more closely aligned with indirect effects more typically aligned with inflammatory soft and hard tissue responses to complex bacterial communities on their surrounding environment (79). Thus, periodontitis is provoked by bacterial biofilms that result in niche activity resulting in tissue inflammation. This inflammatory insult has many wide-reaching and complex effects as seen below (80, 81).

## 4. How landscape ecology can provide a better vision of periodontal disease

Current theory indicates that periodontal diseases are initiated and propagated by an inflammatory reaction to an accumulation of a complex community of biofilm microorganisms that form on tooth surfaces above the gumline initially which then migrate to a region below that dividing line (31, 82). From the point of view of landscape ecology, the disease occurs in the region between the teeth and gum-line and it is reasonable to conclude that the area above the gumline, termed the supragingival area, is the initiating region for inflammatory gingival and periodontal disease (83). As plaque accumulates it moves to the area below the gumline, termed the subgingival area. As mentioned, the supra and subgingival areas are two distinctly different habitats that house different activities and that affect different cells and tissue components (84–86).

The supragingival area is bathed by salivary secretions, has a microbiome that is mostly aerobic, and is exposed to food substances that get masticated and can provide important nutrients to the bacteria that gather as a biofilm on the tooth surface (87). Studies conducted in the 60's and 70's showed that the frequency and type of carbohydrate (liquid, sticky, and retentive) was critical relative to supragingival plaque biofilm development (88). Landmark experiments bt Loe and colleagues illustrated that abstaining from toothbrushing for a three- week period resulted in a consistent inflammatory response at the gingival margin that forms punctate areas of redness and bleeding at the gingival margin (89). Tissue destruction occurs and reverts to health after subjects return to toothbrushing and plaque removal within one week following this experimental gingivitis protocol (89, 90). These studies demonstrated an orderly progression of microbes that form on a clean tooth surface where bacteria move from Gram-positive Streptococcal spp. extending in parallel arrays away from the tooth surface and later transition to a mixture in the parallel interstices that now contain numerous Gram-negative facultative microbes (91). In time, the biofilms become almost equally dominated by aerobic and anaerobic microbes forming a complex microbiome fully extending from the supra to the subgingival domain (92). These simple but dramatic experiments could be altered by requiring these volunteers with health gingiva and teeth to abstain from all oral hygiene for 2 weeks but now also have them rinse with a 10% sucrose solution hourly for 8 h, forcing a microbiome shift. This dietary challenge results in a pH and microbiome change favoring acid loving/acid producing bacteria that form within the biofilm resulting in early carious lesions on the enamel surface. These simple experiments show dramatic examples of how manipulating the environment can reduce fitness for one group of microbes but encourage fitness for another group (Streptococcal spp.) resulting in a different disease outcome (93).

A biofilm developing on a recently cleaned tooth surface occurs in a very specific and orderly progression that encompasses both space and time. Initially saliva forms a pellicle or coating to which pioneer colonizers, namely, Gram-positive Streptococcal spp. attach forming palisading layers of parallel microbes (91, 94). Corynbacterium and Actinomyces follow and a simple aerobic Gram-positive biofilm as time moves on is then infiltrated by a variety of Gram-negative bacteria until a complex climax community is developed (50). Saliva can wash over the plaque and either neutralize the pH, provide nutrients, interfere, or, assist with bacterial survival (2, 48). Thus, saliva is the principal media for tooth surfaces above the gum-line and as such deliver's nutrients to supragingival bacteria (92). Saliva does not find its way below the gumline. In contrast, gingival crevice fluid (GCF), principally a serum exudate, is the main media below the gum-line. Cells (polymorphonuclear leukocytes; PMNs) emanating from blood vessels directly below the gumline pour out into the saliva (95). These fluids bath their respective surfaces and supply host response elements (antibodies, antimicrobials, buffers) to their respective compartments. These two complex fluids can be distinguished from one another (see Table 2). Since the flow of GCF is outward into saliva, components of GCF can be found in saliva. Salivary IgA can be distinguished from GCF because salivary IgA is typically dimerized, has a J-chain that links the two monomeric forms covered by a secretory piece that stabilizes the dimer to resist proteolytic degradation resulting from salivary proteases (87). In contrast serum IgA is monomeric, has no secretory piece and no J chain (77). Overtime the bacteria that are above the gumline migrate to the area below the gumline. Some generalist bacteria such as *Aggregatibacter actinomycetemcomitans* (*Aa*) can live above and below the gumline because they are highly adaptable (83). In contrast, other specialist type microbes do much better below the gum-line [i.e., treponemes, vibrios, bacteroides types; (81–84)].

**Table 2:** Concentration of antibodies and complement in saliva, gingival fluid and serum in mg/100 mL.

	Saliva	Gingival crevice fluid	Serum
IgG	1.4	350	1,250
IgM	0.2	25	80
IgA	19.4	110	220
IgG/IgA ratio	0.07/1	3.2/1	5.7/1
C_3_	0.05	40	150

Modified from Table from reference (77).

### 4.1. Influence of host microenvironment on modulation of community dynamics in periodontitis

Inflammation, the hallmark of periodontitis, can activate a wide range of tissue damage to epithelial cells, cementum, alveolar bone, and collagen in a time dependent manner (96, 97). Damage to these tissues can cause the release of nutrients, components of collagen, iron, and other proteins and small chain fatty acids (98). These host derived elements can provide new environments compatible with further growth of pathobionts in the anerobic periodontal pocket habitat (99).

### 4.2. Influence of the habitat and niche on periodontal diagnosis

The subgingival habitat is a very different than the supragingival habitat. Each habitat is composed of distinct patch or patches that house very different niche types. Serum, the predominant subgingival fluid supplies nutrients such as proteins and fatty acids (100). The inflammatory response to the accumulating subgingival microbiome is intense and clearly directed to prevent invasion of the overwhelming microbial load infringing on the underlying tissue. The dynamic nature of these subgingival microbial interactions is not well elucidated because the habitat is not as readily accessible as the supragingival habitat. However, there are methodologies emerging that can provide a window into this intriguing subgingival habitat.

**Figure 3:**
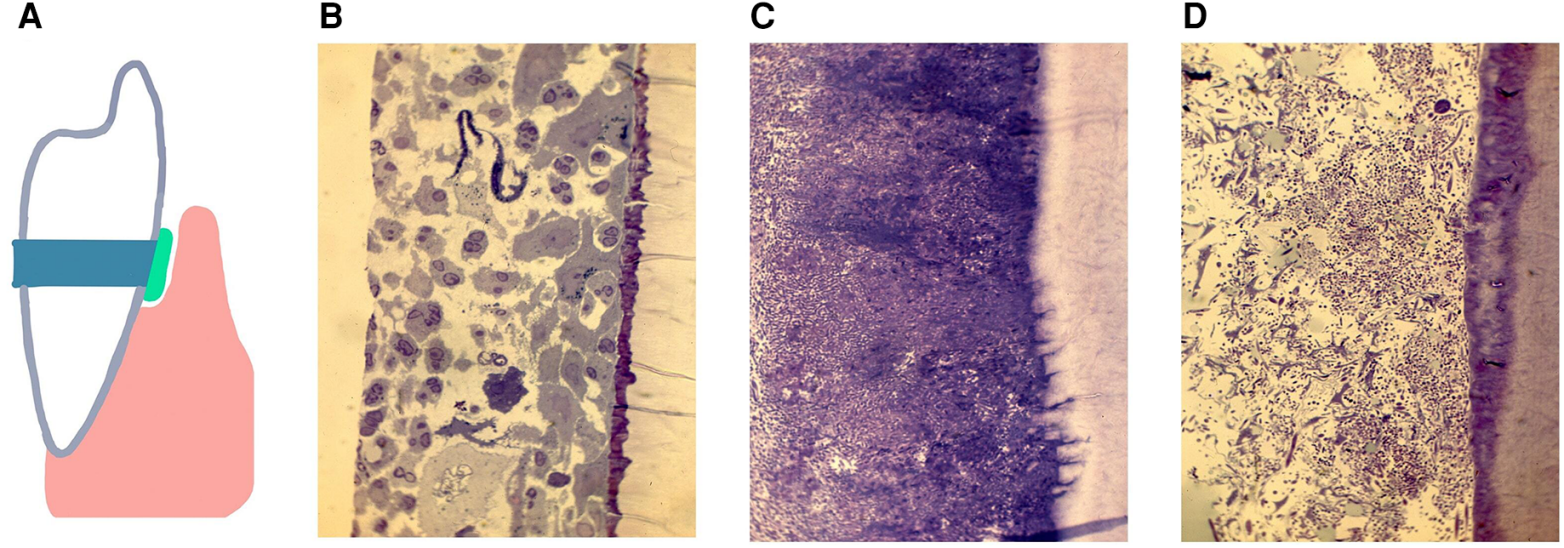
A diagram of a cemental strip tied to the enamel surface, inserted below the gum-line to form a surface for the accumulation of subgingival biofilms (**A**). The pocket was cleaned and debrided, the strip is placed below the gum-line and removed 7 days later, recleaned and a new strip placed and removed 14 or 21 days after placement. The histological specimens show demineralized/epoxy embedded thin sections stained with Toluidine Blue and/or Haematoxylin Eosin. Figures show subgingival plaque on strips collected 14 days after placement and assessed by microscopic observation at 200×s magnification. In pockets from 4 of 5 samples from volunteers with gingivitis. Healthy sites, far left shows epithelium and: PMNs gathering on the strip (**B**). In 6 of 7 samples from patients diagnosed with chronic periodontitis dense plaque was seen with some bacteria penetrating the cemental strip (**C**). In 6 of 7 samples from patients with localized juvenile periodontitis, subgingival plaque levels were minimal as compared to those with chronic periodontitis (**D**). Modified from reference (104).

Methodologies have been introduced that permit study of the subgingival domain. Initially, Transmission Electron Microscopic (TEM) methods were used to study teeth designated for extraction (101). However, these methods suffered from the disadvantage that examination of subgingival plaque biogeography could only be done in a cross-sectional manner, and only gross microscopic identification could be determined (101, 102). Mylar strips were placed subgingivally to assess biofilm development in the subgingival habitat (103) but the mylar surfaces had no relationship to cementum (103). Next cemental strips were acquired from premolar teeth slated for extraction for orthodontic purposes (104). These teeth were cleaned of connective tissue, gamma irradiated for sterilization purposes, cut into 4 strips from one pre-molar, and then placed into pockets that were cleaned. Strips with their microbial contents were removed 7, 14 and 21 days after cleaning. These studies were designed to compare colonization of sites from patients with chronic adult periodontitis (CAP) to those with juvenile localized aggressive periodontitis (LAP) and to those who were “healthy” and had shallow pockets. Microscopic differences were seen as follows, (1) strips from CAP had dense subgingival plaque that often penetrated a demineralized root surface; (2) strips from LAP had a globular deposit on the root surface early on, had significantly less plaque until 14 days after cleaning and had no root penetration, and (3) strips from relatively healthy sites had almost no plaque, had PMNs migrating on either root surfaces or through intercellular spaces on reformed junctional epithelial cells (Figure 3). It was impossible to determine specific microbes using TEM methods. Since then, several methods have shown that microbial identification can be done; first using antibodies directed to the specific usual suspects, then using DNA technologies and most recently and most illuminating using CLASI-FISH which provides a greater potential for defining biogeography (5, 42, 43).

These methods should provide data that will illuminate a better understanding of; (1) who's there, (2) where they are, and (3) how they interact with both the microbial community, and (4) the host response to that challenge. These advanced technologies provide an exciting future for oral microbiologists who are interested in landscape ecology which can lead to a better definition of subgingival niche activities.

### 4.3. Influence of landscape ecology treatment of periodontal disease

Treatment of periodontal disease has had an illustrious history dating back to 1918 when two oral care providers introduced a novel way of using local delivery to retard disease progression. Kritchevsky and Seguin at the Pasteur Institute in Paris introduced the use of neosalvarsan in abscessed pockets that contained fusobacteria and spirochetes (105). Their treatment consisted of local infiltration of a combination of glycerin and 10% neosalvarsan followed by intravenous injections of three low doses of neosalvarsan in addition to a thorough root scaling and polishing (1921). This treatment reduced the presence of the spirochetes as well as the inflammation in the local tissues but at times had adverse side-affects. At some time later Harold Box a prominent scientist and dentist from Toronto introduced a method called oxygen insufflation which was designed to change the subgingival environmental atmosphere from one that was anaerobic to one that was aerobic. While partially successful the substantiveness of this methodology was absent and as such the results were not long lasting (106).

In the late 50's Waerhaug made a point of demonstrating that overhanging restorations posed a serious threat to sustained periodontal health and showed that iatrogenic or poorly performed dentistry can act as a provocative irritant resulting in microbial collections and inflammation in the underlying gingiva (107). The resulting inflammation was due to a challenge to the vasculature directly below the junctional epithelium that caused an exudation of fluid emanating from the underlying vessels and emptying into the gingival crevice and then into the saliva (108). The amount and contents of GCF was dependent on the inflammatory response and could act as a mirror of inflammation in the gingival tissue (109). Work by Page and Schroeder (82) demonstrated that the tissue cellular response could be correlated with inflammation in response to microbial irritants. These classical experiments paved the way toward a better understanding of the challenges of tooth related biofilms to both the supragingival and subgingival space.

One dramatic illustration of this dysfunctional relationship between subgingival biofilm gathering combined with irregular/irritating restorative dental margins was elegantly demonstrated by Lang and associates (110). A restoration was made in the form of a gold inlay that extended across the occlusal surface to both the mesial (front facing side) and distal (back facing side) of a molar tooth. In this experimental model the overhanging restorative margin on the mesial side of the inlay restoration was placed and then the rough irregular surface was allowed to percolate and to collect subgingival plaque for 19 weeks during which time inflammation occurred (110). In conjunction with the inflammation, the subgingival plaque showed an increased growth of Bacteroides app now *Porphyromonas gingivalis* which gained its iron and menadione from the exacerbated bleeding caused by the irritating dental restoration that plunged into the tissue below (110). This irritant caused a tissue response that resulted in inflammation and bleeding and encouraged the overgrowth of Bacteroides type microbes (specialists). The opposite inlay side on the same tooth (the distal surface) had a well-placed smooth margin that resulted in minimal to no inflammation and no Bacteroides spp. To demonstrate the process was marginal overhang/irritant related, the restoration was redesigned, and the experiment was repeated now placing the overhanging margin on the opposite side (distal) of the inlay (110). This new restoration was kept in place for a similar time-period. Once again, the irritant provoked inflammation, bleeding, and a change of the growth pattern in the subgingival microbiota favored overgrowth of *P. gingivalis,* while the opposite side remained healthy. This well-designed experiment demonstrated once again how the environment dictates who lives and thrives (Table 1).

Several less invasive but equally dramatic experiments demonstrate that meticulous and consistent removal of supragingival plaque biofilm influences the subgingival microbiota (111, 112). In the first case consistent and precise removal of supragingival plaque with a toothpick by a researcher from volunteers with 4–6 mm pockets, showed a change in the subgingival pattern of microbes in pockets that were 4 mm in depth but had little effect on pockets that were 6 mm or deeper. We can assume from this modest study that removal of the nutrient continuum produced by members of the supragingival habitat which extends from above the gum-line to below has a range of influence that is limited in terms of its impact on the adjacent subgingival microbiome members. In the second case frequent supragingival scaling described a dramatic effect on reducing “periodontopathic” microorganisms in the subgingival domain in shallow pockets (83). Thus, pocket extension of greater than 3 mm is too great a span for consistent passage of metabolic agents required to support growth of subgingival microorganisms. For this health response related affect to be sustainable, the supragingival cleaning method needs to be repeated over time in a consistent manner (83, 111). These results illustrate that both space and time are effective influencers with regard to patch dynamics in these experiments where subjects already had periodontal pockets (113). Bacteria can alter the environment by induction of inflammation and encouraging overgrowth by inflammophilic pathobionts conducive to fitness for the growth of species that favor continuance of disease. Treatment strategies that are designed to remove these modulating bacterial/inflammophilic forces to restore an environment that favors growth of commensals is a desirable goal.

The sequential development of dental plaque from a clean tooth surface provides a clear demonstration that early tooth colonizers referred to as pioneers have great adaptability that then pave the way for secondary residents that follow in a sequential order (111, 114). Most of these pioneers are commensal/generalist while in their designated habitat. However, these commensal microbes can change in circumstances where tissue ulceration occurs (an over-zealous tooth cleaning in a vulnerable patient). Thus a commensal such as S. sanguis has been shown to exacerbate infective endocarditis when heart valves are infected by *S. sanguis* derived from plaque and deposited on damaged heart valves (115).

Further, recent evidence has shown that *Fusobacterium nulceatum*, a common oral microbe, can move from the oral cavity and lodge onto colo-rectal cancerous lesions and cause an exacerbated reaction at the cancer site. Elimination of these bacteria have been shown to reduce the lesion size (116). In another example, *Aa*, a pathobiont in some cases, can colonize the buccal mucosa but the microbe only becomes relevant when it moves to the tooth surface and then moves into a habitat below the gum-line. These movements from one habitat to another are often related to signals created by the up-regulation of genes provoked by hazardous insults (117). Once in the subgingival domain *Aa* can play a role in periodontitis but it can also move into the blood stream, colonize damaged heart valves in a specific EmA adhesin-receptor interaction and provoke an uptick of infectious endocarditis (118). These examples show how generalist microbes due to their fitness adaptability can be dangerous when they escape their ‘natural” habitat.

**Figure 4:**
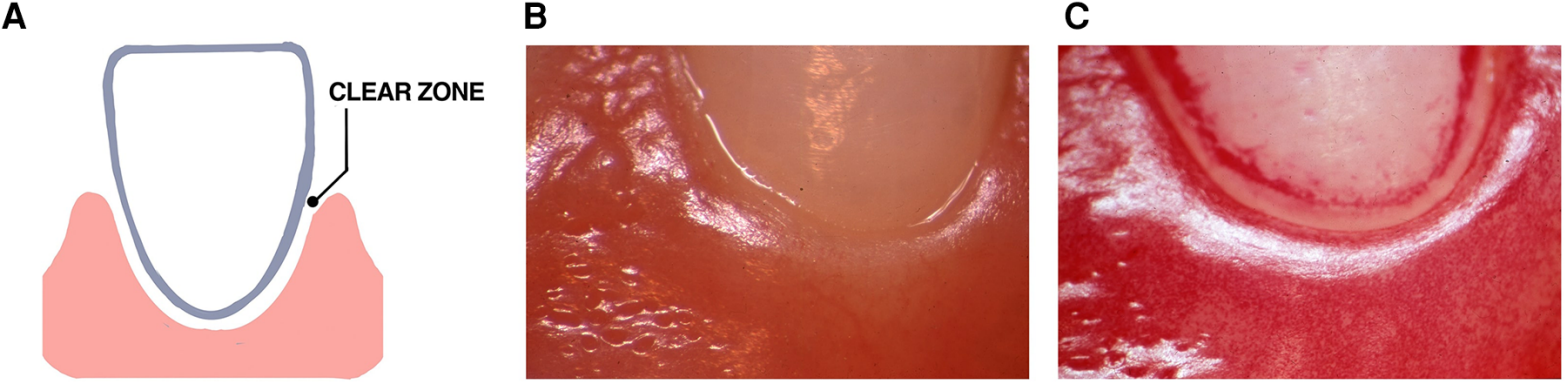
Illustration of the “clear zone” between the gingival margin and forming plaque. A diagram of the “clear zone” shown between the gingival margin and plaque in the experimental gingivitis model (**A**). Stereo-microscopic image showing the absence of plaque at the start of the experimental protocol (**B**). Three days after abstaining from tooth cleaning the “clear zone” is shown in one volunteer (**C**). This “clear zone” was seen in 13 of 16 volunteers assessed in this study. Modified from reference (119).

Many years ago, a “clear zone” was discovered in the area directly above the gingival margin that was observed three days after study participants abstained from all oral hygiene procedures in the experimental gingivitis protocol Figure 4 (119). This zone occurred in 13 of 16 participants, however, was seen to vanish after one day of abstention in three subjects who were considered to be “heavy plaque formers”. In the context of habitat designation, it was unclear whether this zone was due to saliva that accumulated at the gingival margin or whether it was derived from the inflammatory exudate emanating from the vasculature underlying the gingival epithelium. As time went on plaque overcame this “clear zone” and thereafter the plaque biofilm migrated to the area below the gumline (119). In context of landscape ecology in the early stages of biofilm development on a clean tooth surface this “clear zone” could be an important landmark that separates one habitat (supra-gingival) from a second subgingival habitat. Studying the way in which bacteria move from the zone above the gum-line to the area below the gum-line could reveal a transition zone that could alter disease. Questions posed by this mysterious zone can answer whether there are hazardous signals that bacteria in the supragingival zone receive that up-regulate dispersal genes that permit them to move away from their original habitat? Alternatively, is the supragingival habitat too competitive for less hardy bacteria to compete for available metabolic resources?

## 5. Conclusions

This review has presented an overview of landscape ecology and how understanding these ecological principals can be used to enhance our understanding of the oral microbiome.

The review has illustrated how ecological principals have been used in dentistry in the past and how dentistry may benefit from a greater focus on landscape ecology as it intersects with oral biology in the future.

The review also indicates that oral commensal bacteria common to one habitat, the oral cavity, can act more aggressively in a distant habitat and can aggravate disease at distant sites such as the heart, colon, and kidney.

We conclude by stating that the great adaptability of oral bacteria leaves us with the understanding that bacteria where here long before us, will be here long after we have gone and deserve our respect and attention. It suggests to readers that an improved understanding of ecological principals related to bacteria can lead to new diagnostic and therapeutic strategies in our efforts to understand the oral biology and history of “Life Itself”.
